# DHA-Containing Oilseed: A Timely Solution for the Sustainability Issues Surrounding Fish Oil Sources of the Health-Benefitting Long-Chain Omega-3 Oils

**DOI:** 10.3390/nu6052035

**Published:** 2014-05-22

**Authors:** Soressa M. Kitessa, Mahinda Abeywardena, Chakra Wijesundera, Peter D. Nichols

**Affiliations:** 1 CSIRO Animal, Foods and Health Sciences, P.O. Box 10041, Adelaide BC, SA 5000, Australia; E-Mail: Mahinda.Abeywardena@csiro.au; 2 CSIRO Animal, Foods and Health Sciences, Werribee, Victoria, VIC 3030, Australia; E-Mail: Chakra.Wijesundera@csiro.au; 3 Food Futures Flagship, Division of Marine and Atmospheric Research, Hobart, TAS 7000, Australia; E-Mail: Peter.Nichols@csiro.au

**Keywords:** omega-3 long-chain PUFA, aquaculture, alternate sources

## Abstract

Benefits of long-chain (≥C_20_) omega-3 oils (LC omega-3 oils) for reduction of the risk of a range of disorders are well documented. The benefits result from eicosapentaenoic acid (EPA) and docosahexaenoic acid (DHA); optimal intake levels of these bioactive fatty acids for maintenance of normal health and prevention of diseases have been developed and adopted by national and international health agencies and science bodies. These developments have led to increased consumer demand for LC omega-3 oils and, coupled with increasing global population, will impact on future sustainable supply of fish. Seafood supply from aquaculture has risen over the past decades and it relies on harvest of wild catch fisheries also for its fish oil needs. Alternate sources of LC omega-3 oils are being pursued, including genetically modified soybean rich in shorter-chain stearidonic acid (SDA, 18:4ω3). However, neither oils from traditional oilseeds such as linseed, nor the SDA soybean oil have shown efficient conversion to DHA. A recent breakthrough has seen the demonstration of a land plant-based oil enriched in DHA, and with omega-6 PUFA levels close to that occurring in marine sources of EPA and DHA. We review alternative sources of DHA supply with emphasis on the need for land plant oils containing EPA and DHA.

## 1. Introduction

There is a vast array of reviews, systematic reviews, meta-analysis and industry reports on seafood consumption in general and the health benefits of omega*-*3 long-chain (≥C_20_) polyunsaturated fatty acids (omega LC-PUFA) (also termed LC omega-3 oils) in particular. Our review begins from the premise that, as far as human health claims are concerned, the health benefits of seafood and their omega-3 fatty acids are largely related to the supply of eicosapentaenoic acid (EPA, 20:5ω3) and docosahexaenoic acid (DHA, 22:6ω3) [1,2,3,4,5,6,7,8,9]. Thus, one aim of our review is to examine the occurrence of a marked rise in demand for oils high in EPA and DHA and against this demand, the sustainability of marine sources of these LC omega-3 oils. The review summarises recommended intake targets proposed and/or set by national and international bodies. It analyses the bio-conversion of shorter-chain (C_18_) omega-3 fatty acids to EPA and DHA in humans, monogastric animals, ruminants and aquaculture species. As the major user of global fish oil (FO) supply, the aquaculture sector is considered in a relatively greater detail with respect to both biosynthesis of EPA and DHA as well as FO supply issues. Research trends in the area of metabolic engineering of plants to produce novel omega-3 oil sources, and the biological efficacy of currently available oils are covered.

## 2. Consensus for, and Recommendations on, Human Nutrition Needs for Long-Chain Omega-3 Oils

Increasing numbers of clinical and epidemiological studies provide evidence supporting the case that omega-3 LC-PUFA are responsible for a multitude of health benefits [10]. The dietary intake of preformed omega-3 LC-PUFA—EPA and DHA—has been recognised as important [1] since *in vivo* conversion of the shorter-chain (C_18_) fatty acids, namely ω-linolenic acid (ALA, 18:3ω3) to DHA in particular is relatively poor [11,12]. In 2009, the International Society for the Study of Fatty Acids and Lipids (ISSFAL) [13] provided the following position statement: “The majority of evidence from isotopic tracer studies shows that the conversion of ALA to DHA is on the order of 1% in infants, and considerably lower in adults.” Hence, it is critical that there are various and sustainable sources of oils containing preformed DHA for the whole population to meet targets for adequate intake of EPA plus DHA. Table 1 summarises the dietary intake targets proposed by various national and international bodies [14,15,16,17,18,19,20].

Although the recommended daily intakes vary, they are a culmination of years of clinical and epidemiological research that have clearly established a strong body of evidence for the beneficial health effects of LC omega-3 oils for the improvement of cardiovascular health [21]. The recommendations provided in Table 1 are based on consumption of both EPA and DHA. As currently there are only limited and more niche supply alternatives to the marine supply of DHA, we also aim to highlight specific roles of DHA in the following section to caution against reliance on LC omega-3 sources that do not provide DHA.

**Table 1:** Selected suggested long-chain (LC) omega-3 (eicosapentaenoic acid + docosahexaenoic acid (EPA + DHA)) intakes (mg/day) for adults available from various agencies and bodies.

Group	EPA + DHA, mg/day
SACN/COT, UK 2004 [14]	450
National Heart Foundation (Australia), 2008 [15]	500
American Dietetic Association and Dieticians of Canada, 2007 [16]	500
FAO/WHO Expert Consultation, 2008 [17]	250–2000 *
American Heart Association, 2002 [18]:	
Coronary heart disease sufferers	1000
Those seeking to reduce triacylglycerols (blood fats)	2000–4000
National Health and Medical Research Council (Australia) [19]:	
Male adults	610
Female adults	430
European Food Safety Authority, 2010 [20]	250

* For secondary prevention of coronary heart disease.

## 3. The Role of DHA

LC omega-3 oils containing EPA and DHA are considered beneficial for certain aspects of cardiovascular health and pharmaceutical-grade omega-3 LC-PUFA therapies have expanded rapidly for treatment of cardiovascular-related diseases [22,23,24]. Whilst the majority of studies have reported both EPA and DHA as being protective, there is a growing body of evidence that suggests differential effects depending on the nature of cardiovascular risk factor itself and/or the disease endpoint. For example, DHA has been more effective than EPA for its actions on blood pressure, heart rate and vascular health [10,25,26,27]. On their effects on plasma lipids, a meta-analysis of randomized placebo controlled trials of EPA or DHA monotherapy has concluded that DHA is more effective in lowering triacylglycerols (TAG) and raising HDL-cholesterol (HDL-c) than EPA [28]. Although DHA has been found to raise LDL-cholesterol (LDL-c), this was also associated with increased LDL and HDL particle sizes [29]—an outcome not observed with EPA [26]. Furthermore, only DHA was effective in reducing the number of small, dense LDL particles [27] which are known to be more atherogenic. Increased LDL and HDL particle sizes are negatively correlated with cardiovascular risk. Furthermore, DHA, but not EPA, was inversely associated with intima-media thickness—an independent predictor of cardiovascular events—in the Japanese [30], suggesting more potent anti-atherogenic properties of DHA. Similarly, DHA but not EPA supplementation reduced the vulnerability to experimentally-induced atrial fibrillation and secondary structural changes (re-modeling) of the atria [31]; these findings are in agreement with observations made in several human clinical studies that reported that lower incidence of atrial fibrillation is correlated positively with plasma DHA, but not EPA [32,33].

The human nervous system contains a significant amount of DHA, which is required for brain development and function especially in infants [12]. With the poor conversion of ALA and EPA to DHA, together with the particularly important roles for DHA in humans, inclusion of DHA in infant formula is now widespread.

With regard to mental health conditions, a range of studies have examined the effect of the LC omega-3 oils on mild cognitive impairment (MCI) [34]. Depressive symptoms may increase the risk of progressing from MCI to dementia. Consumption of LC omega-3 may alleviate both cognitive decline and depression. A recent study investigated the benefits of DHA and EPA supplementation for depressive symptoms, quality of life (QOL) and cognition in elderly people with MCI [34]. In a 6-month, double-blind, randomised controlled trial, individuals aged 65 years with MCI were allocated to receive a supplement rich in EPA, DHA or the omega-6 PUFA linoleic acid (LA, 18:2ω6). Compared with the LA group, Geriatric Depression Scale (GDS) scores improved in the EPA and DHA groups and verbal fluency (Initial Letter Fluency) improved in the DHA group. Improved GDS scores were correlated with increased DHA plus EPA. Improved self-reported physical health was associated with increased DHA. There were no treatment effects on other cognitive or QOL parameters. Increased intakes of DHA and EPA benefited mental health in older people with MCI. Increasing omega-3 LC-PUFA intakes may reduce depressive symptoms and the risk of progressing to dementia. The authors concluded that this needs to be investigated in larger, depressed sample groups with MCI.

The same research team also examined the effects of LC omega-3 oils on literacy and behaviour in children with attention-deficit/hyperactivity disorder (ADHD) [35]. The effects of an EPA-rich oil and a DHA-rich oil *versus* an omega-6 PUFA-rich safflower oil (control)—as LA—were compared in a randomized, controlled trial. The effect of supplementation on cognition, literacy, and parent-rated actions was assessed by linear mixed modelling. There were no significant differences between the supplement groups in the primary outcomes after four months. However, the erythrocyte fatty acid profiles indicated that an increased proportion of DHA was associated with improved word reading and lower parent ratings of oppositional behaviour. These effects were more evident in a subgroup of children with learning difficulties: an increased erythrocyte DHA was associated with improved word reading, improved spelling, an improved ability to divide attention, and lower parent ratings of oppositional behaviour, hyperactivity, restlessness, and overall ADHD symptoms. The authors concluded that increases in erythrocyte omega-3 PUFA, specifically DHA, may improve literacy and conduct in children with ADHD. The greatest benefit may be observed in children who have co-morbid learning difficulties. In a recent randomized controlled intervention trial, DHA supplementation was observed to improve both memory and reaction time in healthy young adults whose habitual diets were low in DHA [36]; the response was found to be modulated by gender.

Another very recent application of DHA has been in the development of neuroprotective strategies for treatment of spinal cord and head injuries. These studies—albeit at early stages involving animal models—illustrate the significant potential of DHA, but not EPA, in the treatment of acute neurological injury [37].

Against the increasing scientific literature pointing to the importance of the LC omega-3 oils and in particular DHA, in human health, global supplies of fish oil (the current main source of EPA and DHA) obtained from wild-harvest low-value marine species, termed forage fish, will not meet future market demands [23,38]. The following sections in this review paper examine sustainability of fish oil and future sources of the LC omega-3 oils, recent findings for the use of SDA containing oils, current practices with aquafeeds, and further research needs.

## 4. Supply, Demand and Environmental Issues—A Need for Alternative Sources of LC Omega-3 Oils

The harvest of low trophic species such as anchovy, sardines, mackerel, menhaden, capelin and sandeel for the production of fish meal and fish oil (FO) represents the current main source of the health-benefiting LC omega-3 oils used in aqua and animal feeds, health supplements, pharmaceuticals and other products including functional foods.

Fish oil processing involves a range of steps after the initial meal and oil production [39]. Many of these steps use the same processes that are used for vegetable oils. The final use for the oil determines the level of processing, with human nutrition and pharmaceutical applications generally requiring greater processing and accompanying quality assurance and quality control procedures than for fish and animal nutrition. Several changes in the usage pattern have occurred over the past decades. FO was initially widely used in livestock feed, then, as aquaculture expanded this industry became the major user. Therapeutic uses of LC omega-3 PUFA are increasingly being recognized and recently new pharmaceutical grade LC omega-3 products (containing 85%–95% EPA and DHA) have gained increasing market share, and this industry has expanded its share of use of the fish oil resource, with less oil available for the aquaculture sector [31,40]. This changing pattern of use of FO is predicted to continue [40]. One aspect of this recent change is that the processing of the FO to achieve the higher grade (or more pure) LC omega-3 products results in considerable losses, with product yields in the 5%–10% range. For instance, in a purification process reported by Belarbi *et al.* [41], production of 1 kg EPA ester required 15 kg of FO, or 56 kg (dry basis) of the marine alga *Phaeodactylum tricornutum* or 70 kg of another alga *Monodus subterraneus*. Further research and development for more efficient production of the pharmaceutical grade products will assist all users to maximize and better utilize this important finite resource.

Global production of FO is around 1 million tonnes per annum, with fish meal in the range of 6–7 million tonnes per annum, except during the periodic El Niño years [42]. Production has generally remained at this level for the past decade [23], and requires an annual catch of 25–30 million tonnes of feed-grade fish and unwanted fish processing waste; 4–5 kg of wet fish yields 1 kg of FO and fishmeal. Although the current harvest of low trophic (also termed forage) species for fish meal and FO has been regarded as sustainable for several decades, a recent development has been the foreseen need to reduce the harvest of small oceanic forage fish like sardines and anchovies in some areas by 20%–50% in order to protect larger predators that rely on these species for food [43,44]. Should such recommendations be implemented, this would have significant flow-on ramifications for the range of industries currently utilizing the FO resources.

An emerging source of LC omega-3 oils over the past decade has been krill oil. The current krill harvest is around 200,000 MT [40], and is actually much less than in the 1980s prior to the break-up of the USSR. The total allowable catch is set by the Committee for the Conservation of Antarctic Marine Living Resources (CCAMLR) and is three times greater than the present harvest. Of the major krill oil producers, a further development has been that Aker BioMarine has been granted certification by the Marine Stewardship Council (MSC) in 2012. MSC has validated the harvesting and traceability for Aker’s Antarctic fisheries. The total krill harvest is deemed sustainable at present levels in view of a number of, although not all, bodies including various Non-Government Organizations. If there is to be a major expansion of the krill fishery, the sustainability topic will clearly need to be revisited by these and other expert groups including in particular CCAMLR.

## 5. Alternative Sources of LC Omega-3 Oils

Against the background provided above on the current and future status of marine-derived oils—fish oil and more recently krill oil—considerable progress has occurred with the development of new, alternate and sustainable sources of the LC omega-3 oils. Single cell organisms (SCO), such as heterotrophic dinoflagellates and thraustochytrids (both grown and harvested for DHA containing oils) and other algal groups and recently a genetically modified (GM) yeast (containing EPA) are now in commercial production; strong uptake has occurred for the DHA oils in particular areas including infant formula, health supplements and some functional foods [45]. In addition to this excellent progress with SCO production of the LC omega-3 oils, a large number of groups are conducting research and development with a suite of microalgae towards co-production of biofuels and other by-products [46]. Whilst the cost of production of microalgae for biofuel production is presently greater than for fossil fuels, future breakthroughs in culturing, harvesting and other processing are anticipated [46], which can be expected to reduce costs. It is anticipated that these uses for SCO will largely remain for the high value applications including in nutraceutical and pharmaceuticals rather than in aquafeeds.

**Figure 1:**
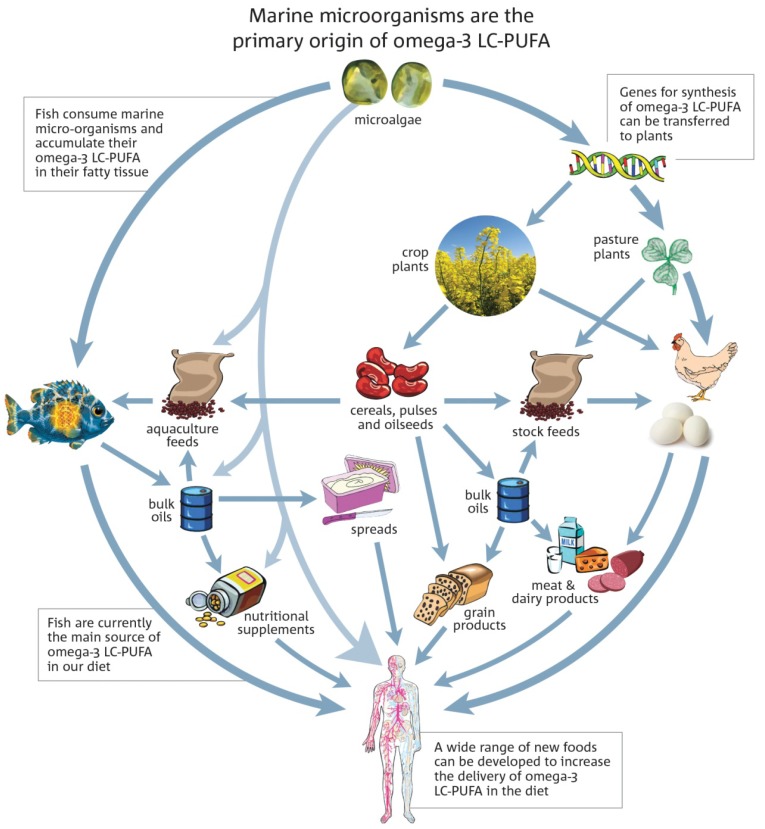
The potential future sources of omega-3 LC-PUFA are shown, with current sources (left) being seafood and microalgae, with possible future sources through genetically engineered plants also indicated at the right.

The past decade has also seen several groups using genetic engineering to allow oilseed crops to produce LC omega-3 oils [23]. As this research field has progressed, important breakthrough steps have included: the isolation and characterization of genes from the marine microalgae encoding front-end desaturases involved in DHA biosynthesis [47], the isolation of highly efficient desaturases and elongases [48,49,50,51], the use of genes with omega-3 substrate preference [49,50,51] and the development and use of a land plant (tobacco) leaf-based assay using interchangeable design principles to rapidly assemble multistep recombinant pathways [52]. Progress with research on insertion of microalgal-derived genes leading to DHA production into a range of omega-3 C_18_ PUFA accumulating land plants has been reviewed [2,53,54,55]. Transfer of genes from microorganisms to land plants has seen accumulation in oilseeds of SDA, EPA and DHA [2] (Table 2, Figure 1). Good progress has been made in engineering the EPA genes into crop plants, with several groups reporting the production of EPA at levels similar to that observed in bulk fish oil (approximately 18%) [56,57]. The conversion of the C_20_ EPA to the particularly important C_22_ DHA, however, had been problematic with many attempts resulting in the accumulation of EPA and DPA, but until very recently little DHA [56,58,59,60,61,62].

For SDA and EPA, levels achieved in the engineered oilseed plants are comparable to levels from other naturally occurring land plant (SDA) and/or marine (EPA) sources [23]. Elevated levels of DHA had not been achieved prior to 2012, except for one *in planta* observation where the isolated TAG fraction from the leaf of *Nicotiana benthemiana* contained high DHA, although high levels of the omega*-*6 PUFA 18:2ω6 were also present [53]. In 2012, a further key breakthrough occurred, with the reporting, for the first time, of fish oil-like profiles for a DHA-containing oilseed plant *Arabidopsis thaliana* [58]. Features of the new oil were: (i) a DHA level of 15% (of the total FA); (ii) a total of 25% new omega-3 PUFA and omega-3 LC-PUFA; (iii) 30% ALA; and (iv) an omega-3/omega-6 ratio that was similar to that observed for marine oils. The latter feature is a further important and distinguishing attribute for the land plant derived LC omega-3 oils, with this report being, to our knowledge, the first time this oil trait has been observed. More recently a similar profile has been observed in a commercial oilseed plant (*Camelina sativa*) [59].

**Table 2:** Levels of SDA, EPA and DHA (as % of fatty acids) in new land plant oil seeds.

Oil Seed and Comparison to *Farmed salmon*	Reference	SDA%	EPA%	DHA%
CSIRO Oil Seeds (includes model plants)	[51]	10		
	[59]		5	1
	[51]	1	26	
	[53]		15	14
	[58]	5	2	15
	[59]	9	3	13
BASF Mustard	[61]		15	1.5
Monsanto-Soy	[56]	20		
Dupont-Soy	[55,57]		20	3
***Farmed salmon***				
fed fish oil (FO)	[63,64]		10	17
fed plant oil/chicken fat-FO	[65]		1–5	5

Abbreviations: SDA, stearidonic acid EPA; EPA, eicosapentaenoic acid; DHA, docosahexaenoic acid.

These findings clearly indicate the feasibility of developing oilseed crops with high concentrations of omega-3 LC-PUFA. Further research and development is needed to develop commercial oilseed crops with LC omega-3 oil enriched in DHA as has been achieved in model plants (*Arabidopsis,* tobacco) and also in *Camelina*. Future novel land-based plants can provide an economically viable source of LC omega-3 oil for aquaculture and other higher value applications. A land plant source of LC omega-3, if achieved, and assuming cultivation is permitted, will be considerably cheaper than that from microorganisms, and could be used as an additional source of these essential ingredients in feed, food and pharmaceutical products to improve human health (Figure 1).

## 6. ALA and SDA in Animal Feeds

**Figure 2:**
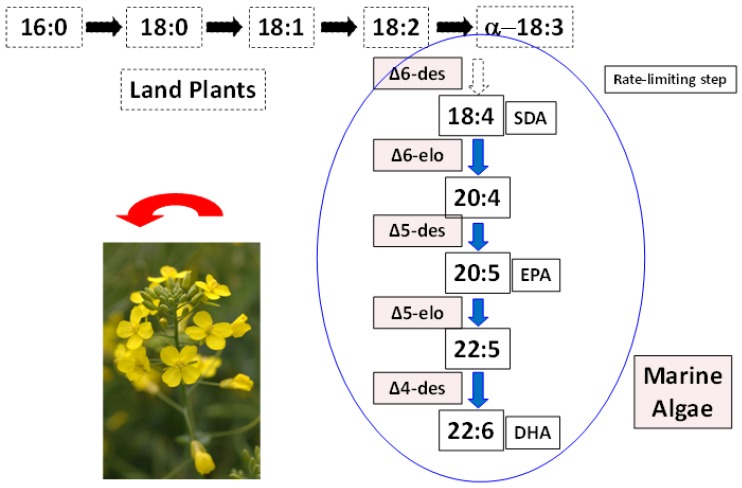
Schematic showing synthesis of shorter-chain fatty acids in land plants (**black horizontal arrows**), followed by addition of genes from marine microalgae (**blue vertical arrows**) resulting in new LC omega-3 containing oilseeds. elo, elongase; des, desaturase.

The availability of the SDA-containing *Echium* oil (around 14% SDA of total FA) has enabled this potential EPA/DHA precursor, which is one step more advanced than ALA to be trialed in animal, including farmed fish, and human nutrition research. The general hypothesis driving this line of research has been that animals and farmed fish fed SDA oils would produce tissue containing greater proportion of EPA and DHA than those fed ALA oils. This is based on the assumption that the inefficiency in the biosynthesis of EPA and DHA from ALA is related to the rate-limiting step of converting ALA to SDA (Figure 2).

**Figure 3:**
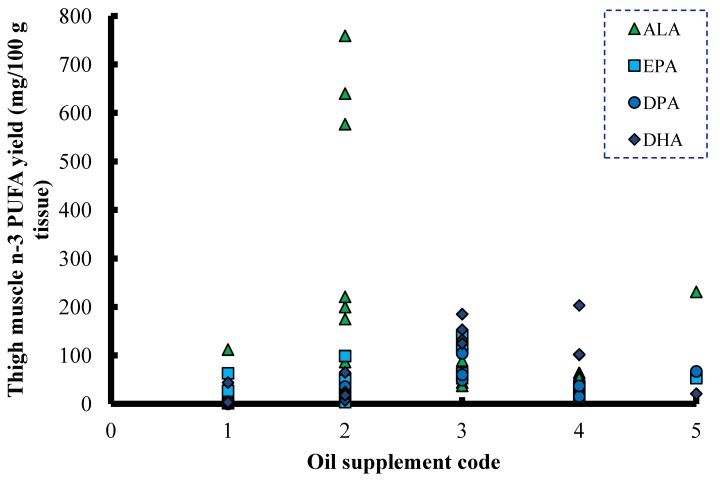
Levels of omega-3 PUFA (ALA) and omega-3 LC-PUFA (EPA, DPA and DHA) in thigh muscle from broilers on a diet with: (1) no oil supplement; (2) vegetable oil; (3) fish oil; (4) marine algae; or (5) SDA-containing oil [66,67,68,69,70,71,72,73,74].

**Figure 4:**
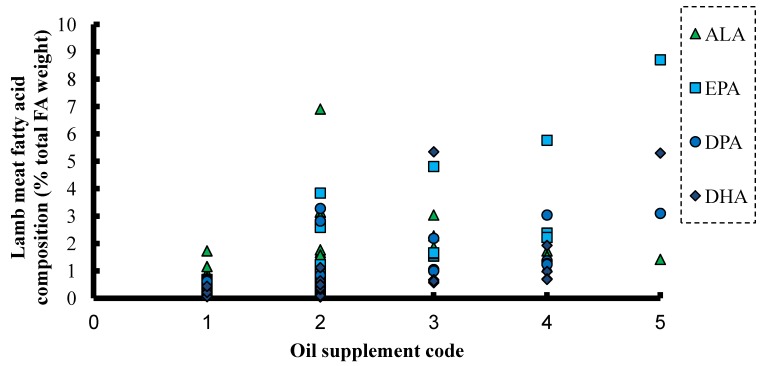
Levels of omega-3 PUFA (ALA) and omega-3 LC-PUFA (EPA, DPA and DHA) in trimmed lean muscle of lambs on a diet with: (1) no oil supplement; (2) linseed or linseed oil; (3) fish oil–vegetable oil mix; (4) fish oil; or (5) fish oil–marine algae mix [75,76,77,78,79,80,81,82,83,84].

Selected poultry data from feeding experiments [66,67,68,69,70,71,72,73,74] where different oils have been used to enrich thigh muscle are summarised in Figure 3. There were only modest changes in EPA and DHA content in broiler muscle samples when the oil supplement itself did not contain the omega-3 LC-PUFA. The evidence from such a large range of experiments did not indicate marked benefit from using C_18_ oils in enriching tissues particularly with DHA (Figure 3). Similar trends were noted when we reviewed EPA and DHA enrichment of egg from omega-3 oil-supplemented laying hens and breast muscle in broilers (data not shown). Similarly, data from lamb meat studies [75,76,77,78,79,80,81,82,83,84] are summarized in Figure 4. Levels of EPA and DHA in lamb meat were lowest in studies where vegetable oils were used. As the dietary fat supplement shifted towards marine sources, the levels of EPA and DHA in lamb meat increased across experiments. The evidence so far suggests that the best way to enhance the DHA content of livestock products is to include DHA containing fat supplements in the diet. There is as yet no convincing evidence that current fat supplements containing ALA or SDA are suitable alternatives for those containing preformed EPA and especially DHA.

## 7. ALA and SDA in Aquafeeds

Aquaculture continues to be the main end user of global sources of FO, yet available sources of FO generally remain static and will likely not increase significantly. Against this background of either static or decreasing resource availability, the demand for FO and its range of uses continues to increase as noted earlier. The increasing demand for FO is in line with the growing global population (due to rise by a further 34% by 2050) [85], the expanding aquaculture industry (around 10% growth per annum), the increasing recognition of the health benefits of the LC omega-3 oils, and more recently the resulting and therefore competing use of FO for production of pharmaceutical grade products containing ≥85% EPA and/or DHA.

A number of trials have been conducted for Atlantic salmon and barramundi using the SDA rich *Echium* oil *(Echium plantagineum)*. Initial work with Atlantic salmon parr (freshwater stage) showed that SDA was effective in producing EPA and DHA [86,87]. This freshwater phase is only a short period of the total life cycle for farmed Atlantic salmon. Trials for the same species during the seawater stage (bulk of the life cycle) showed SDA was not so effective [63,64,88]. Some EPA was produced and also DPA, but no DHA. The same observations occurred for barramundi feeding trials [89,90,91], that is limited or no omega-3 LC-PUFA, in particular DHA, was produced or accumulated in the flesh of this species. Conversion of the C_18_ SDA for barramundi to the omega-3 LC-PUFA was even lower than that observed for Atlantic salmon. Other researchers have generally observed similar findings where SDA inclusion has occurred in aquafeed trials with other fish species, including Atlantic cod, striped bass, rainbow trout and gilthead seabream [92,93,94,95].

A further feeding trial with the SDA-containing *Echium* oil for early juvenile barramundi used the plant bioactive sesamin as a potential modulator of lipid biosynthesis [96]. Relative to the control fish, growth of the SDA treatment group was hindered, although interestingly both EPA and DHA increased. It was proposed that sesamin is a potent modulator for LC-PUFA biosynthesis in barramundi, but probably will have more effective impact at advanced ages. By modulating certain lipid metabolic pathways, the use of sesamin as a feed ingredient probably disrupted the body growth and development of organs and tissues in the early juvenile barramundi.

Atlantic salmon and barramundi have, when fed an FO-containing diet, provided an excellent source of beneficial omega-3 LC-PUFA for human consumption, but reduced concentrations of these acids, together with a markedly decreased omega-3/omega-6 ratio, as occurs through the use of vegetable oil and/or animal fat diets, may compromise their nutritional benefit to consumers. Limited research has been performed to examine this issue. In one study [97], dietary intake of differently fed salmon (100% FO, 50/50 FO/rapeseed oil, 100% rapeseed oil) and the influence on markers of human atherosclerosis were compared. Significant differences between the consumer groups were observed in the serum fatty acid profiles, especially for the levels of total omega-3 PUFA and the omega-3/omega-6 ratio, which were markedly increased in the FO-fed fish consuming group in contrast to the two other groups. The authors concluded that Atlantic salmon fed the FO-containing diet and containing very high concentrations of omega-3 LC-PUFA seemed to impose favorable biochemical changes in patients with CHD when compared with ingestion of fillets with intermediate and low levels of the marine omega-3 LC-PUFA, where FO was replaced in part or in full by rapeseed oil [93]. There have been no consumer trials with fish fed diets containing ALA / SDA rich oils *versus* FO derived EPA + DHA, and looking at the effects on consumers.

## 8. SDA Oils in Animal Models and Humans

It is apparent from examination of the research performed to date on SDA diets, that the benefits from use of SDA (like for ALA) are due to its conversion to EPA and DHA. Whilst SDA is more efficiently converted to EPA than ALA [98], it is important to record that the elongation of dietary SDA to DHA has been found to be absent (or negligible at best) in humans [99,100,101]. Furthermore, with respect to enrichment with EPA, the conversion efficiency of SDA to EPA was only 17% even after four months of feeding soybean oil preparation containing 16% SDA and 11% ALA. Similarly, three months treatment with soybean oil with even higher SDA content (28% SDA alone and 40% total omega-3) failed to change erythrocyte DHA from baseline values. These studies clearly show the inability of SDA-rich oils to influence the endogenous DHA pool in humans. The elongation and desaturation of SDA appears to terminate at the DPA level as increased levels of this fatty acid have been observed [101]. However, evidence of direct physiological benefits of DPA in humans is yet to be elucidated. The accumulation of DPA in the EPA and DHA biosynthesis pathways of many species, and its relative abundance in red meat and some marine species also point to a need to determine whether or not DPA should be included in the omega-3 content claim of foods and ingredients. Inclusion of DPA will broaden the range of foods that can reach the “good source” and “very good source” bars in the omega-3 content claims.

Similar results have also been found following animal feeding studies where dietary *Echium* oil rich in SDA (and ALA; 15% SDA, 29% ALA) failed to increase plasma or tissue DHA levels compared to supplementation with fish oil. In particular, *Echium* oil diet did not lead to any increase in EPA or DHA in cardiac muscle membranes, but resulted in a dose-related increase in DPA [102]. Fish oil feeding on the other hand displayed considerable accumulation of DHA but not DPA. Also it is of interest to note that anti-arrhythmic action (protection against ischemia induced cardiac arrhythmia and sudden cardiac death in rats) was significantly greater following feeding with FO compared to SDA-rich *Echium* oil [65].

Albert *et al.* [103] reported over a decade ago that plasma LC omega-3 levels are inversely associated with the risk of sudden cardiac death. More recently, the omega-3 index which is the combined total proportion of erythrocyte membrane EPA and DHA has emerged as a novel biomarker that predicts cardiovascular risk [104]. It has also been reported that the omega-3 index correlates well with the EPA + DHA levels of myocardial membranes [105], thus favouring its use when assessing the risk of sudden cardiac death. Since there was no increase in DHA following feeding oils rich in SDA [99,100,101], the reported beneficial rise in omega-3 index has been driven solely by an increase in EPA. In view of the overall cardiovascular benefits specific to DHA, including the anti-arrhythmic actions discussed above, it can be concluded that further research is needed to determine the important question whether or not an omega-3 index that increased solely due to EPA, is of less benefit as compared to rise in omega-3 index achieved via greater incorporation of DHA into erythrocytes, and ultimately whether an increased SDA consumption would translate into improved human health outcomes [101]. Taken collectively, the observations summarized in these two sections covering the potential use of SDA-rich oils presently imply that direct supply of the pre-formed omega-3 LC-PUFA (EPA and in particular DHA) is the preferred strategy to improve the omega-3 status in diverse applications ranging from aquaculture, livestock and in humans.

## 9. Current Practices with Commercial Aquafeeds and Future Sources of LC Omega-3 Oils

**Figure 5:**
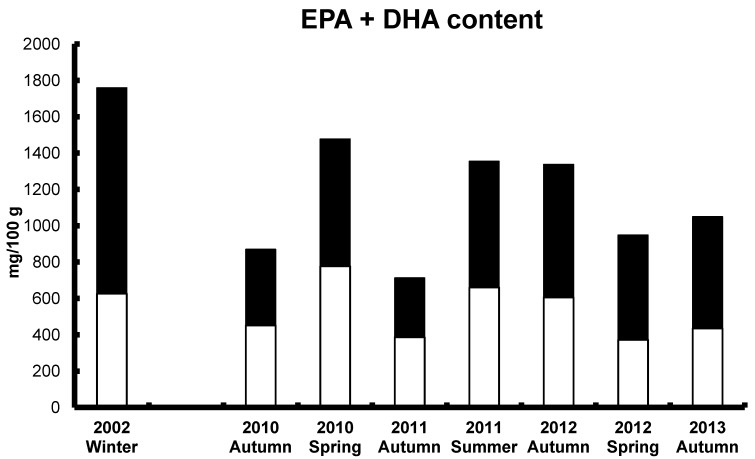
Farmed Atlantic salmon from Tasmania, Australia from 2002 (fish oil diet) [23] and 2010 to 2013 (chicken fat/fish oil diet) [108]: Content of EPA (**white bars**) and DHA (**black bars**) (mg/100 g, wet weight).

Aquaculture can be considered as a traditional industry with fish culture occurring for many centuries. Modern aquaculture expansion began in the 1980s and has continued to rise steadily since, with the high value salmonoid fish such as Atlantic salmon being the species of choice. Fish oil, produced as a by-product of the fish meal industry, had been the main oil incorporated into fish feed until recent years. As noted earlier, the past decade has seen fish oil availability decrease and also prices increase substantially. From the use of 100% FO (of the oil component), feed manufacturers are now using up to 75% or higher of vegetable or animal-derived oils [106]. The topic of FO replacement and alternative lipid sources has been recently examined in considerable detail, with a review book now available for researchers, industry and other end users [107]. Given the availability of such a substantial resource, it is not the purpose of this section to further review this topic. This FO substitution can include mixes such as FO/rapeseed oil, FO/chicken fat, and also other combinations. Whilst fish growth and performance is generally not affected, the concentration of omega-3 LC-PUFA and the omega-3/omega-6 ratio in fillet products is markedly changed. For farmed Atlantic salmon grown in Tasmania, Australia, the concentration of EPA and DHA has reduced by ≥30%–50% or more (Figure 5), and the omega-3/omega-6 ratio also has reduced markedly. Marine fish typically show an omega-3/omega-6 ratio of between 5 and 10, and for the first time in 2013 the ratio in farmed salmon in Australia has decreased to less than 1 [108].

A recent study tested whether Atlantic salmon smolt fed a diet with a higher DHA: EPA ratio and a lower content of LC omega-3 oils to that of conventional FO based diets would enhance deposition of LC omega-3 in the liver and muscle [109]. Comparisons were made between fish fed: (1) a FO diet; (2) a blend of 50% rapeseed and 50% tuna oil diet (termed model oil, MO1); (3) a blend of 50% rapeseed, 25% tuna and 25% FO diet (MO2); and (4) a blend of 50% FO and 50% chicken fat diet (FO/CF). The latter diet was representative of commercial diets in use at the time of the study, with the proportion of chicken fat increasing even further since the study was performed. The dietary DHA:EPA ratio was in the order MO1 > MO2 > FO/CF ~ FO. The LC omega-3 content was approximately 2-fold lower in the MO1, MO2 and FO/CF diets compared to the FO diet, with the relative levels (as % total FA) lowest in the MO1 diet. For the feeding trial, there were comparable contents of LC omega-3 in the muscle of the FO, MO1 and FO/CF fed fish. A major outcome was that a higher DHA:EPA ratio than that commonly occurring with FO-only diets used for Atlantic salmon was better suited for more efficient deposition of LC omega-3, in particular DHA, with evidence therefore apparent for LC omega-3 “sparing” in Atlantic salmon smolts when fed a diet with a high DHA:EPA ratio [109].

The use of a 50% FO and 50% CF blend in aquafeeds for Atlantic salmon, as was in the range commercially practiced in Australia, resulted in comparable LC omega-3 content in the muscle [108] and liver of juvenile Atlantic salmon to a FO fed fish. It is noteworthy that such an oil blend decreases the inefficient utilization of a 100% FO diet, due to the high loss of EPA in particular, and can be considered as an appropriate current strategy, in terms of LC omega-3 sparing, for present use in aquafeeds for Atlantic salmon [108]. It is important to note that in spite of changes that have occurred in feeding practices, farmed Tasmanian Atlantic salmon still remains one of the best sources of omega-3 LC-PUFA oils available to Australian consumers. However, the scope can exist with the potential future use of new oilseed-derived LC omega-3 to restore the content of these health-benefitting ingredients, and also the omega-3/omega-6 ratio to that previously seen.

Further research is needed to determine the optimum relative and absolute concentrations of dietary EPA and DHA to enhance their deposition in larger-sized commercially farmed Atlantic salmon. The rationale to pursue such studies will be reliant on research in plant genomics since oils with the desired FA profiles, in particular containing a high DHA:EPA ratio [58,59], whilst not presently available, will likely be a commercial reality by the end of this decade.

## 10. TAG Structure of Plant-Based LC Omega-3 Oils for Optimum Bioactivity and Food Processing

The melting characteristic of a fat/oil is an important determinant of its suitability for the manufacture of food products. For example, a certain melting range is required before a fat can be used for the manufacture of margarine or fat spreads. The positional distribution of omega-3 LC-PUFA within the TAG molecules can significantly influence the melting characteristics of LC omega-3 oils. In general, the melting point is increased when the omega-3 LC-PUFA is located at the *sn*-2 position compared with the *sn*-1(3) positions [110]. This has practical implications enabling the conversion of liquid oils to semi-solid fats for margarine manufacture or use as *trans* fat substitutes in bakery products. Furthermore, omega-3 LC-PUFA such as DHA are more resistant to oxidative deterioration when located at the *sn*-2 position compared to the *sn*-1(3) positions [111].

The effects of fatty acid positional distribution on absorption and nutrition of oils and fats are less well understood. Evidence from animal and human infant studies suggests that TAG structure affects digestibility, atherogenicity and fasting lipid levels, with fats containing palmitic and stearic acid in the *sn*-2 position being better digested and considered more harmful for cardiovascular health [112,113,114]. However, a few studies in human adults have indicated that fatty acid positional distribution has no effect on digestibility or fasting plasma lipids [115,116]. There have been very limited studies on the physiological effects of TAG positional distribution of omega-3 LC-PUFA such as EPA and DHA on either animals or humans. These fatty acids are predominantly located at the *sn*-2 position in fish oil TAG with the notable exception of seal blubber oil. Though it has been hypothesised, there are presently not sufficient data with humans to conclude that location of omega-3 LC-PUFA at the *sn-*2 position confers greater physiological benefit when compared to location at the *sn-*1 *or sn*-3 positions. We consider this as an area for further fruitful research including with animal model and clinical trials. The available evidence does not yet support a preference as to how the LC omega-3 containing TAG in novel oilseeds should be best assembled to maximize the nutritional benefits to human consumers. As we acquire this knowledge, the prospect of tailoring the DHA positional distribution of novel plant-based DHA oils (both during metabolic engineering and post-harvest) can be used to meet optimum health and food processing properties 

## 11. Conclusions

The need for further clinical trials to better refine our understanding of the mode of action of the health-benefitting LC omega-3 oils will continue, including with emphasis towards the mode of action of the individual components namely EPA and DHA. Similar requirements exist for farmed species, in particular cultured fish. The latter are being increasingly fed non-marine oils, although limited research has occurred on the possible deleterious effects to the farmed species of the lower dietary proportions of LC omega-3 oils, accompanied by a lower omega-3/omega-6 ratio, and ultimately to human consumers. The issue of the finite supply of the fish oil resource is very clearly upon us, and new sustainable sources of these oils are required. SCO derived oils are in use, although remain, and likely will remain, relatively expensive and therefore better suited to niche applications. The past five years have seen expanded interest in applications with krill oil, although considerable care with exploitation of this environmentally sensitive and important resource must occur. After several decades of research for production of LC omega-3 oils from oil seeds, the prospects for such a supply are now a reality, including most recently the difficult to achieve yet nutritionally important DHA. LC omega-3 oils derived from GM oil seed crops may in the future provide the most economically viable source of these key essential ingredients for aquaculture and a range of other applications. It is estimated that the cost and availability of oils from GM plants would be similar to that of currently available commercial oilseed crops such as rapeseed and soya. Further research and development in this area has the potential for significant commercial, health, social and environmental benefits. This exciting field of research will now move into the commercial development phase, with feeding and other trials and associated approvals and consumer acceptance to occur. Other areas of research for continuing effort will include: improved processing and yields for pharmaceutical grade products, improvement and/or further development of novel delivery modes for application of LC omega-3 oils in functional foods, examination of the effects of omega-3 LC-PUFA positional distribution on the bioactivity and processing properties of various LC omega-3 food products, and also for the omega-3 index to gain increasing acceptance and use. Collectively research and development in these and other areas will ensure that enhanced intake of the LC omega-3 oils can occur for a wider range of consumers, with resultant global heath, economic and social benefits resulting.

## References

[1] Calder P.C., Yaqoob P. (2012). Marine omega-3 fatty acids and coronary heart disease. Curr. Opin. Cardiol..

[2] Greene J., Ashburn B.A., Razzouk L., Smith D.A. (2013). Fish oils, coronary heart disease, and the environment. Am. J. Public Health.

[3] Kimmig L.M., Karalis D.G. (2013). Do omega-3 polyunsaturated fatty acids prevent cardiovascular disease? A review of the randomized clinical trials. Lipid Insights.

[4] Roncaglioni M.C., Tombesi M., Avanzini F., Barlera S., Caimi V., Longoni P., Marzona I., Milani V., Silletta M.G., Tognoni G. (2013). *n*-3 Fatty acids in patients with multiple cardiovascular risk factors. N. Engl. J. Med..

[5] Cleland L.G., Caughey G.E., James M.J., Proudman S.M. (2006). Reduction of cardiovascular risk factors with longterm fish oil treatment in early rheumatoid arthritis. J. Rheumatol..

[6] Proudman S.M., Cleland L.G., James M.J. (2008). Dietary omega-3 fats for treatment of inflammatory joint disease: Efficacy and utility. Rheum. Dis. Clin. N. Am..

[7] Stall L.A., Begg D.P., Weisinger R.S., Sinclair A.J. (2008). The role of omega-3 fatty acids in mood disorders. Curr. Opin. Investig. Drugs.

[8] Jeffrey B.G., Weisinger H.S., Neuringer M., Mitchell D.C. (2001). The role of docosahexaenoic acid in retinal function. Lipids.

[9] Parletta N., Milte C.M., Meyer B.J. (2013). Nutritional modulation of cognitive function and mental health. J. Nutr. Biochem..

[10] Ruxton C.H.S., Reed S.C., Simpson M.J.A., Millington K.J. (2007). The health benefits of omega-3 polyunsaturated fatty acids: A review of the evidence. J. Hum. Nutr. Diet..

[11] Williams C.M., Burdge G. (2006). Long-chain *n*-3 PUFA: Plant V. marine sources. Pro. Nutr. Soc..

[12] Abeywardena M.Y., Patten G.S. (2011). Role of ω3 long-chain polyunsaturated fatty acids in reducing cardio-metabolic risk factors. Endocr. Metab. Immune Disord. Drug Targets.

[13] Brenna J.T., Salem N., Sinclair A.J., Cunnane S.C. (2009). α-Linolenic acid supplementation and conversion to *n*-3 long chain polyunsaturated fatty acids in humans. Prostaglandins Leukot. Essent. Fatty Acids.

[14] Scientific Advisory Committee on Nutrition (SACN) and Committee on Toxicity (COT) (2004). Advice on Fish. Consumption: Benefits and Risks.

[15] National Heart Foundation Australia Position Statement: FISH, Fish Oils, *n*-3 Polyunsaturated Fatty Acids and Cardiovascular Health. Updated November 2008. Pro-067, 2nd Edition. National Heart Foundation of Australia. http://www.heartfoundation.org.au/healthy-eating/Pages/fish-oil-program.aspx.

[16] American Dietetics Association and Dietitians of Canada (2007). Position of the American Dietetics Association and Dietitians of Canada: Dietary fatty acids. J. Am. Diet. Assoc..

[17] FAO/WHO Interim summary of conclusions and dietary recommendations on total fat and fatty acids. Proceedings of Joint FAO/WHO Expert Consultation on Fats and Fatty Acids in Human Nutrition.

[18] Kris-Etherton P.M., Harris W.S., Appel L.J., American Heart Association Committee (2002). Fish consumption, fish oil, omega-3 fatty acids and cardiovascular disease. Circulation.

[19] NHMRC (2006). Nutrient Reference Values for Australia and New Zealand.

[20] European Food Safety Authority (2010). Scientific Opinion on Dietary Reference Values for fats, including saturated fatty acids, polyunsaturated fatty acids, monounsaturated fatty acids, *trans* fatty acids, and cholesterol. EFSA Panel on Dietetic Products, Nutrition, and Allergies (NDA). EFSA J..

[21] Leaf A. (2008). Historical overview of *n*-3 fatty acids and coronary heart disease. Am. J. Clin. Nutr..

[22] Bays H.E. (2007). Safety considerations with omega-3 fatty acid therapy. Am. J. Cardiol..

[23] Nichols P.D., Petrie J., Singh S. (2010). Long-chain omega-3 oils—An update on sustainable sources. Nutrients.

[24] Swanson D., Block R., Mousa S.A. (2012). Omega-3 fatty acids EPA and DHA: Health benefits throughout life. Adv. Nutr..

[25] Mori T.A., Woodman R.J. (2006). The independent effects of eicosapentaenoic acid and docosahexaenoic acid on cardiovascular risk factors in humans. Curr. Opin. Clin. Nutr. Metab. Care.

[26] Cottin S.C., Sanders T.A., Hall W.L. (2011). The differential effects of EPA and DHA on cardiovascular risk factors. Proc. Nutr. Soc..

[27] Kelley D.S., Adkins Y. (2012). Similarities and differences between the effects of EPA and DHA on markers of atherosclerosis in human subjects. Proc. Nutr. Soc..

[28] Wei M.Y., Jacobson T.A. (2011). Effects of eicosapentaenoic acid *versus* docosahexaenoic acid on serum lipids: A systematic review and meta-analysis. Curr. Athereoscler. Rep..

[29] Neff L.M., Culliner J., Cunnigham-Rundles S., Seidman C., Meehan D., Maturi J., Wittkowski K.M., Levine B., Breslow J.L. (2011). Algal docosahexaenoic acid affects plasma lipoprotein particle size distribution in overweight and obese adults. J. Nutr..

[30] Sekikawa A., Kadowaki T., El-Saed A., Okamura T., Sutton-Tyrrell K., Nakamura Y., Evans R.W., Mitsunami K., Edmundowicz D., Nishio Y. (2012). Differential association of docosahexaenoic and eicosapentaenoic acids with carotid intima-media thickness. Stroke.

[31] Ramadeen A., Connelly K.A., Leong-Poi H., Hu X., Fujii H., Laurent G., Domenichiello A.F., Bazinet R.P., Dorian P. (2012). Docosahexaenoic acid, but not eicosapentaenoic acid, supplementation reduces vulnerability to atrial fibrillation. Circ. Arrhythm. Electrophysiol..

[32] Wu J.H., Lemaitre R.N., King I.B., Song X., Sacks F.M., Rimm E.B., Heckbert S.R., Siscovick D.S., Mozaffarian D. (2012). Association of plasma phospholipid long-chain ω-3 fatty acids with incident atrial fibrillation in older adults: The cardiovascular health study. Circulation.

[33] Virtanen J.K., Mursu J., Voutilainen S., Tuomainen T.P. (2009). Serum long-chain *n*-3 polyunsaturated fatty acids and risk of hospital diagnosis of atrial fibrillation in men. Circulation.

[34] Sinn N., Milte C.M., Street S.J., Buckley J.D., Coates A.M., Petkov J., Howe P.R. (2012). Effects of *n*-3 fatty acids, EPA v. DHA, on depressive symptoms, quality of life, memory and executive function in older adults with mild cognitive impairment: A 6-month randomised controlled trial. Br. J. Nutr..

[35] Milte C.M., Parletta N., Buckley J.D., Coates A.M., Young R.M., Howe P.R. (2012). Eicosapentaenoic and docosahexaenoic acids, cognition, and behavior in children with attention-deficit/hyperactivity disorder: A randomized controlled tria. Nutrition.

[36] Stonehouse W., Conlon C.A., Podd J., Hill S.R., Minihane A.M., Haskell C., Kennedy D. (2013). DHA supplementation improved both memory and reaction time in healthy young adults: A randomized controlled trial. Am. J. Clin. Nutr..

[37] Hall J.C.E., Priestley J.V., Perry V.H., Michael-Titus A.T. (2012). Docosahexaenoic acid, but not eicosapentaenoic acid, reduces the early inflammatory response following compression spinal cord injury in the rat. J. Neurochem..

[38] Tacon A.G.J., Hasan M.R., Metian M. (2008). Demand and supply of feed ingredients for farmed fish and crustaceans: Trends and prospects.

[39] Bimbo A.P., Hernandez E.M., Hosokawa M. (2011). Production of marine oils. Omega-3 Oils. Applications in Functional Foods.

[40] Naylor R.L., Hardy R.W., Bureau D.P., Chiu A., Elliott M., Farrell A.P., Forster I., Gatlin D.M., Goldburg R.J., Hua K. (2009). Feeding aquaculture in an era of finite resources. Proc. Natl. Acad. Sci. USA.

[41] Belarbi E.H., Molina E., Chisti Y. (2000). A process for high yield and scaleable recovery of high purity eicosapentaenoic acid esters from microalgae and fish oil. Enzyme Microb. Technol..

[42] Barlow S.M. (2000). Fish meal and fish oil: Sustainable feed ingredients for aquafeeds. Global Aquac. Advoc..

[43] Smith A.D.M., Brown C.J., Bulman C.M., Fulton E.A., Johnson P., Kaplan I.C., Lozano-Montes H., Mackinson S., Marzloff M., Shannon L.J. (2011). Impacts of fishing low-trophic level species on marine ecosystems. Science.

[44] Pikitch E., Boersma P.D., Boyd I.L., Conover D.O., Cury P., Essington T., Heppell S.S., Houde E.D., Mangel M., Pauly D. (2012). Little Fish, Big Impact: Managing a Crucial Link in Ocean. Food Webs.

[45] Barclay W., Weaver C., Metz J., Hansen J., Cohen Z., Ratledge C. (2010). Development of a docosahexaenoic acid production technology using *Schizochytrium*: Historical perspective and update. Single Cell Oils, Microbial and Algal Oils.

[46] Lam M.K., Lee K.T. (2012). Microalgae biofuels: A critical review of issues, problems and the way forward. Biotechnol. Adv..

[47] Zhou X.-R., Robert S.S., Petrie J.R., Frampton D.M., Mansour M.P., Blackburn S.I., Nichols P.D., Green A.G., Singh S.P. (2007). Isolation and characterization of genes from the marine microalga *Pavlova salina* encoding front-end desaturases involved in docosahexaenoic acid biosynthesis. Phytochemistry.

[48] Robert S.S., Petrie J.R., Zhou X.-R., Mansour M.P., Blackburn S.I., Green A.G., Singh S.P., Nichols P.D. (2009). Isolation and characterisation of a Δ5-fatty acid elongase from the marine microalga *Pavlova salina*. Mar. Biotechnol..

[49] Petrie J.R., Liu Q., Mackenzie A.M., Shrestha P., Mansour M.P., Robert S.S., Frampton D.F., Blackburn S.I., Nichols P.D., Singh S.P. (2010). Isolation and characterisation of a high-efficiency desaturase and elongases from microalgae for transgenic LC-PUFA production. Mar. Biotechnol..

[50] Petrie J.R., Shrestha P., Liu Q., Mansour M.P., Wood C.C., Zhou X.R., Nichols P.D., Green A.G., Singh S.P. (2009). Rapid expression of transgenes driven by seed-specific constructs in leaf tissue: DHA production. Plant Methods.

[51] Petrie J.R., Shrestha P., Mansour M.P., Nichols P.D., Liu Q., Singh S.P. (2010). Metabolic engineering of omega-3 long-chain polyunsaturated fatty acids in plants using an acyl-CoA Δ6-desaturase with ω3-preference from the marine microalga *Micromonas pusilla*. Metab. Eng..

[52] Wood C.C., Petrie J.R., Shrestha P., Mansour M.P., Nichols P.D., Green A.G., Singh S.P. (2009). A leaf-based assay using interchangeable design principles to rapidly assemble multistep recombinant pathways. Plant. Biotechnol. J..

[53] Petrie J.R., Singh S.P. (2011). Expanding the docosahexaenoic acid food web for sustainable production: Engineering lower plant pathways into higher plants. AoB Plants.

[54] Qi B.X., Fraser T., Mugford S., Dobson G., Sayanova O., Butler J., Napier J.A., Stobart A.K., Lazarus C.M. (2004). Production of very long chain polyunsaturated omega-3 and omega-6 fatty acids in plants. Nat. Biotechnol..

[55] Venegas-Caleron M., Sayanova O., Napier J.A. (2010). An alternative to fish oils: Metabolic engineering of oil-seed crops to produce omega-3 long chain polyunsaturated fatty acids. Prog. Lipid Res..

[56] Kinney A.J., Cahoon E.B., Damude H.G., Hitz W.D., Kolar C.W., Liu Z.-B. (2004). Production of very Long Chain Polyunsaturated Fatty Acids in Oilseed Plants. International Patent Application.

[57] Cheng B., Wu G., Vrinten P., Falk K., Bauer J., Qiu X. (2010). Towards the production of high levels of eicosapentaenoic acid in transgenic plants: The effects of different host species, genes and promoters. Transgenic Res..

[58] Petrie J.R., Shrestha P., Zhou X.-R., Mansour M.P., Liu Q., Belide S., Nichols P.D., Singh S.P. (2012). Metabolic engineering plant seeds with fish oil-like levels of DHA. PLoS One.

[59] Petrie J.R., Shrestha P., Belide S., Kennedy Y., Lester G., Liu Q., Divi U.K., Mulder R.J., Mansour M.P., Nichols P.D. (2014). Metabolic engineering *Camelina sativa* with fish oil like levels of DHA. PLoS One.

[60] Robert S.S., Singh S.P., Zhou X.-R., Mansour M.P., Liu Q., Belide S., Nichols P.D., Singh S.P. (2005). Metabolic engineering of *Arabidopsis* to produce nutritionally important DHA in seed oil. Funct. Plant. Biol..

[61] Wu G., Truksa M., Datla N., Vrinten P., Bauer J., Zank T., Cirpus P., Heinz E., Qiu X. (2005). Stepwise engineering to produce high yields of very long-chain polyunsaturated fatty acids in plants. Nat. Biotechnol..

[62] Finney A.J. (2006). Metabolic engineering in plants for human health and nutrition. Curr. Opin. Biotechnol..

[63] Miller M.R., Bridle A.R., Nichols P.D., Carter C.G. (2008). Increased elongase and desaturase gene expression with stearidonic acid enriched diet does not enhance long-chain (*n*-3) content of seawater Atlantic salmon (*Salmo salar* L.). J. Nutr..

[64] Codabaccus M.B., Bridle A.R., Nichols P.D., Carter C.G. (2011). Effect of feeding Atlantic salmon (*Salmo salar* L.) a diet enriched with stearidonic acid from parr to smolt on growth and *n*-3 LC-PUFA biosynthesis. Br. J. Nutr..

[65] Abeywardena M.Y., Wijesundera C. (2013).

[66] Rymer C., Gibbs R.A., Givens D.I. (2010). Comparison of algal and fish sources on the oxidative stability of poultry meat and its enrichment with omega-3 polyunsaturated fatty acids. Poult. Sci..

[67] Rymer C., Hartnell G.F., Givens D.I. (2011). The effect of feeding modified soybean oil enriched with C18:4*n*-3 to broilers on the deposition of *n*-*3* fatty acids in chicken meat. Br. J. Nutr..

[68] Poureslami R., Raes K., Huyghebaert G., de Smet S. (2010). Effects of diet, age and gender on the polyunsaturated fatty acid composition of broiler anatomical compartments. Br. Poult. J..

[69] Yang X., Zhang B., Guo Y., Jiao P., Long F. (2010). Effects of dietary lipids and *Clostridium butyricum* on fat deposition and meat quality of broiler chickens. Poult. Sci..

[70] Jia W., Rogiewicz A., Bruce H.L., Slominski B.A. (2010). Feeding flaxseed enhances deposition of omega-3 fatty acids in broiler meat portions in different manner. Can. J. Anim. Sci..

[71] Lopez-Ferrer S., Baucells M.D., Barroeta A.C., Gashorn M.A. (1999). *n*-3 Enrichment of chicken meat using fish oil: Alternative substitution with rapeseed and linseed oils. Poult. Sci..

[72] Lopez-Ferrer S., Baucells M.D., Barroeta A.C., Gashorn M.A. (2001). *n*-3 Enrichment of chicken meat. 1. Use of very long-chain fatty acids in chicken diets and their influence on meat quality: Fish oil. Poult. Sci..

[73] Roth-Maier D.A., Eder K., Kirchgessner M. (1998). Liver performance and fatty acid composition of meat in broiler chickens fed diets with various amounts of ground or whole flaxseed. J. Anim. Physiol. Anim. Nutr..

[74] Schreiner M.H.W., Hulan H.W., Razzazi-Fazeli E., Böhm J., Moreira R.G. (2005). Effect of different sources of dietary omega-3 fatty acids on general performance and fatty acid profiles of thigh, breast liver and portal blood of broilers. J. Sci. Food Agric..

[75] Bas P., Berthelot V., Pottier E., Normand J. (2007). Effect of level of linseed on fatty acid composition of muscles and adipose tissues of lambs with emphasis on *trans* fatty acids. Meat Sci..

[76] Nute G.R., Richardson R.J., Wood J.D., Hughes S.I., Wilkinson R.G., Cooper S.L., Sinclair L.A. (2004). Effect of dietary oil source on the flavour and colour and lipid stability of lamb meat. Meat Sci..

[77] Kitessa S.M., Gulati S.K., Ashes J.R., Scott T.W., Fleck E. (2001). Effect of feeding tuna oil supplement protected against hydrogenation in the rumen on growth and *n*-3 fatty acid content of lamb fat and muscle. Aus. J. Agric. Res..

[78] Kitessa S.M., Williams A., Gulati S., Boghossian V., Reynolds J., Pearce K.L. (2009). Influence of duration of supplementation with ruminally protected linseed oil on the fatty acid composition of feedlot lambs. Anim. Feed Sci. Technol..

[79] Kitessa S., Liu S.M., Briegel J., Pethick D., Gardner G., Ferguson M., Allingham P., Nattrass G., McDonagh M., Eric Ponnampalam E. (2010). Effects of intensive or pasture finishing in spring and linseed supplementation in autumn on the omega-3 content of lamb meat and its carcass distribution. Anim. Prod. Sci..

[80] Jerónimo E.E., Alves S.P., Prates J.A.M., Santos-Silva J., Bessa R.J. (2009). Effect of dietary replacement of sunflower oil with linseed oil on intramuscular fatty acids of lamb meat. Meat Sci..

[81] Diaz M.T., Caneque V., Sanchez C.I., Lauzurica S., Perez C., Fernandez C., Alvarez I., de la Fuente J. (2011). Nutritional and sensory aspects of light lamb meat enriched in *n*-3 fatty acids during refrigerated storage. Food Chem..

[82] Berthlot V., Bas P., Pottier E., Normand J. (2012). The effect of maternal linseed supplementation and/or lamb linseed supplementation on muscle and subcutaneous adipose tissue fatty acid composition of indoor lambs. Meat Sci..

[83] Wachira A.M., Sinclair L.A., Wilkinson G., Enser M., Wood J.D., Fisher A.V. (2002). Effects of dietary fat source and breed on the carcass composition, *n*-3 polyunsaturated fatty acid and conjugated linoleic acid content of sheep meat and adipose tissue. Br. J. Nutr..

[84] Elmore J.S., Cooper S.L., Enser M., Mottram D.S., Sinclair A.L., Wilkinson R.G., Wood J.D. (2005). Dietary manipulation of fatty acid composition in lamb meat and its effect on the volatile aroma compounds of grilled lamb. Meat Sci..

[85] FAO How to Feed the World in 2050. http://www.fao.org.

[86] Miller M.R., Nichols P.D., Carter C.G. (2007). Replacement of dietary fish oil with a stearidonic acid containing oil for Atlantic salmon (*Salmo salar* L.). Comp. Biochem. Physiol..

[87] Miller M.R., Nichols P.D., Carter C.G. (2008). Omega-3 oil sources for use in aquaculture—Alternatives to the unsustainable harvest of wild fish. Nutr. Res. Rev..

[88] Codabaccus B.M., Bridle A.R., Nichols P.D., Carter C.G. (2011). An extended feeding history with a stearidonic acid enriched diet from parr to smolt increases *n*-3 long-chain polyunsaturated fatty acids biosynthesis in white muscle and liver of Atlantic salmon (*Salmo salar* L.). Aquaculture.

[89] Tu W.C., Muhlhausler B.S., James M.J., Stone D.A., Gibson R.A. (2013). Dietary alpha-linolenic acid does not enhance accumulation of omega-3 long-chain polyunsaturated fatty acids in barramundi (*Lates calcarifer*). Comp. Biochem. Physiol..

[90] Alhazzaa R., Bridle A.R., Nichols P.D., Carter C.G. (2011). Replacing dietary fish oil with *Echium* oil enriched barramundi with C_18_ PUFA rather than long-chain PUFA. Aquaculture.

[91] Alhazzaa R., Bridle A.R., Nichols P.D., Carter C.G. (2011). Up-regulated desaturase and elongase gene expression promoted accumulation of polyunsaturated fatty acid (PUFA) but not long-chain PUFA in *Lates calcarifer*, a tropical euryhaline fish, fed a stearidonic acid- and γ-linoleic acid-enriched diet. J. Agric. Food Chem..

[92] Bell J.G., Strachan F., Good J.E., Tocher D.R. (2006). Effect of dietary echium oil on growth, fatty acid composition and metabolism, gill prostaglandin production and macrophage activity in Atlantic cod (*Gadus morhua* L.). Aquac. Res..

[93] Bharadwaj A.S., Hart S.D., Brown B.J., Li Y., Watkins B.A., Brown P.B. (2010). Dietary source of stearidonic acid promotes higher muscle DHA concentrations than linolenic acid in hybrid striped bass. Lipids.

[94] Cleveland B.C., Francis D.S., Turchini G.M. (2012). Echium oil provides no benefit over linseed oil for (*n*-3) long-chain PUFA biosynthesis in rainbow trout. J. Nutr..

[95] Diaz-Lopez M., Perez M.J., Acosta N.G., Tocher D.R., Jerez S., Lorenzo A., Rodriguez C. (2009). Effect of dietary substitution of fish oil by echium oil on growth, plasma parameters and body lipid composition in gilthead seabream (*Sparus aurata* L.). Aquac. Nutr..

[96] Alhazzaa R., Bridle A.R., Carter C.G., Nichols P.D. (2012). Sesamin modulation of lipid class and fatty acid profile in early juvenile teleost, *Lates calcarifer*, fed different dietary oils. Food Chem..

[97] Seierstad S.L., Poppe T.T., Koppang E.O., Svindland A., Rosenlund G, Frøyland L., Larsen S. (2005). Influence of dietary lipid composition on cardiac pathology in farmed Atlantic salmon, *Salmo salar* L. J. Fish. Dis..

[98] James M.J., Ursin V.M., Cleland L.G. (2003). Metabolism of stearidonic acid in human subjects: Comparison with the metabolism of other *n*-3 fatty acids. Am. J. Clin. Nutr..

[99] Harris W.S., Lemke S.L., Hansen S.N., Goldstein D.A., di Rienzo M.A., Su H., Nemeth M.A., Taylor M.L., Ahmed G., George C. (2008). Stearidonic acid-enriched soybean oil increased the omega-3 index, an emerging cardiovascular risk marker. Lipids.

[100] Lemke S.L., Vicini J.L., Su H., Goldstein D.A., Nemeth M.A., Krul E.S., Harris W.S. (2010). Dietary intake of stearidonic acid-enriched soybean oil increases the omega-3 index: randomized, double-blind clinical study of efficacy and safety. Am. J. Clin. Nutr..

[101] Walker C.G., Jebb S.A., Calder P.C. (2013). Stearidonic acid as a supplemental source of ω-3 polyunsaturated fatty acids to enhance status for improved human health. Nutrition.

[102] Abeywardena M.Y., Kitessa S., Nichols P.D. Dietary *Echium* oil rich in stearidonic (18:4ω3) acid does not increase cardiac membrane EPA or DHA. Proceedings of the Nutrition Society of Australia, 34th Annual meeting.

[103] Albert C.M., Campos H., Stampfer M.J., Ridker P.M., Manson J.E., Willett W.C., Ma J. (2002). Blood levels of long-chain *n*-3 fatty acids and the risk of sudden death. N. Engl. J. Med..

[104] Harris W.S., von Schacky C. (2004). The omega-3 index: A new risk factor for death from coronary heart disease?. Prev. Med..

[105] Harris W.S., Sands S.A., Windsor S.L., Ali H.A., Stevens T.L., Magalski A., Porter C.B., Borkon A.M. (2004). Omega-3 fatty acids in cardiac biopsies from heart transplantation patients: Correlation with erythrocytes and response to supplementation. Circulation.

[106] Smullen R. (2013). Personal communication.

[107] Turchini G.M., Ng W.K., Tocher D. (2010). Fish Oil Replacement and Alternative Lipid Sources in Aquaculture Feeds.

[108] Nichols P.D., Petrie J.P., Singh S.P. (2014). Readily available sources of long-chain omega-3 oils: Is farmed Australian seafood a better source of the good oil than wild-caught seafood?. Nutrients.

[109] Codabaccus B.M., Carter C.G., Bridle A.R., Nichols P.D. (2012). The “*n*-3 LC-PUFA sparing effect” of modified dietary *n*-3 LC-PUFA content and DHA to EPA ratio in Atlantic salmon smolt. Aquaculture.

[110] Fraser B., Perlmutter P., Wijesundera C. (2007). Practical synthesis of triacylglycerol regioisomers containing long-chain polyunsaturated fatty acids. J. Am. Oil Chem. Soc..

[111] Wijesundera C., Ceccato C., Watkins P., Fagan P., Fraser B., Thienthong N., Perlmutter P. (2008). Docosahexaenoic acid is more stable to oxidation when located at the *sn*-2 position of triacylglycerol compared to *sn*-1(3). J. Am. Oil Chem. Soc..

[112] Hayes K.C. (2001). Synthetic and modified glycerides: Effects on plasma lipids. Curr. Opin. Lipidol..

[113] Sundram K., Karupaiah T., Hayes K.C. (2007). Stearic acid-rich interesterified fat and *trans*-rich fat raise the LDL/HDL ratio and plasma glucose relative to palm olein in humans. Nutr. Metab..

[114] Robinson D.M., Martin N.C., Robinson L.E., Ahmadi L., Marangoni A.G., Wright A.J. (2009). Influence of interesterification of a stearic acid-rich spreadable fat on acute metabolic risk factors. Lipids.

[115] Hunter J.E. (2001). Studies on effects of dietary fatty acids as related to their position on triglycerides. Lipids.

[116] Berry S.E.E. (2009). Triglycerol structure and interesterification of palmitic and stearic acid-rich fats: An overview and implications for cardiovascular disease. Nutr. Res. Rev..

